# Long-term follow-up of *Mycoplasma hyopneumoniae*-specific immunity in vaccinated pigs

**DOI:** 10.1186/s13567-023-01145-1

**Published:** 2023-03-01

**Authors:** Evelien Biebaut, Lisa Beuckelaere, Filip Boyen, Freddy Haesebrouck, Charles-Oliver Gomez-Duran, Bert Devriendt, Dominiek Maes

**Affiliations:** 1 Department of Internal Medicine, Reproduction and Population Medicine, Faculty of Veterinary Medicine, Ghent University, Salisburylaan 133, 9820 Merelbeke, Belgium; 2 Department of Pathobiology, Pharmacology and Zoological Medicine, Faculty of Veterinary Medicine, Ghent University, Salisburylaan 133, 9820 Merelbeke, Belgium; 3 Department of Translational Physiology, Infectiology and Public Health, Faculty of Veterinary Medicine, Ghent University, Salisburylaan 133, 9820 Merelbeke, Belgium; 4 Boehringer Ingelheim Vetmedica, Binger Strasse 173, 55216 Ingelheim, Germany

**Keywords:** *Mycoplasma hyopneumoniae*, pigs, longitudinal study, vaccine-induced immunity, cell-mediated immunity

## Abstract

*Mycoplasma hyopneumoniae* is the primary agent of enzootic pneumonia in pigs. To minimize the economic losses caused by this disease, *M. hyopneumoniae* vaccination is commonly practiced. However, the persistence of *M. hyopneumoniae* vaccine-induced immunity, especially the cell-mediated immunity, till the moment of slaughter has not been investigated yet. Therefore, on two commercial farms, 25 pigs (*n* = 50) received a commercial bacterin intramuscularly at 16 days of age. Each month, the presence of *M. hyopneumoniae*-specific serum antibodies was analyzed and the proliferation of and TNF-α, IFN-γ and IL-17A production by different T cell subsets in blood was assessed using recall assays. Natural infection with *M. hyopneumoniae* was assumed in both farms. However, the studied pigs remained *M. hyopneumoniae* negative for almost the entire trial. Seroconversion was not observed after vaccination and all pigs became seronegative at two months of age. The kinetics of the T cell subset frequencies was similar on both farms. *Mycoplasma hyopneumoniae*-specific cytokine-producing CD4^+^CD8^+^ T cells were found in blood of pigs from both farms at one month of age but decreased significantly with increasing age. On the other hand, T cell proliferation after in vitro *M. hyopneumoniae* stimulation was observed until the end of the fattening period. Furthermore, differences in humoral and cell-mediated immune responses after *M. hyopneumoniae* vaccination were not seen between pigs with and without maternally derived antibodies. This study documents the long-term *M. hyopneumoniae* vaccine-induced immune responses in fattening pigs under field conditions. Further research is warranted to investigate the influence of a natural infection on these responses.

**Supplementary Information:**

The online version contains supplementary material available at 10.1186/s13567-023-01145-1.

## Introduction

One of the most important respiratory pathogens in pigs is *Mycoplasma hyopneumoniae (M. hyopneumoniae*). It is the primary agent of enzootic pneumonia, endemically present on most farms and responsible for significant economic losses in the pig production worldwide [1, 2]. Vaccination is the most commonly used management practice to control *M. hyopneumoniae*, since it can minimize the clinical signs and performance losses, and reduce treatment costs [3]. The goal of *M. hyopneumoniae* vaccination is to raise pathogen-specific immune responses which respond fast and effectively upon a natural *M. hyopneumoniae* infection. Most commercially available *M. hyopneumoniae* vaccines for pigs are registered for administration between one and three weeks of age [4]. The onset of *M. hyopneumoniae*-specific immunity varies from one to four weeks and aims to protect against infections during the complete fattening period, until slaughter [4].

Overall, vaccination or infection of pigs triggers the differentiation of memory B cells and CD4^+^CD8^+^ T cells, ensuring a fast antibody and cell-mediated immune response after a second contact with the pathogen [5]. Vaccination against *M. hyopneumoniae* may result in seroconversion and an increase in *M. hyopneumoniae*-specific antibodies locally in the respiratory tract [6–9]. In vaccinated animals, higher levels of IFN-γ-producing lymphocytes were observed together with a higher lymphocyte proliferation rate after in vitro *M. hyopneumoniae* stimulation compared to non-vaccinated animals [9–11]. Furthermore, polyfunctional CD4^+^CD8^−^ and CD4^−^CD8^+^ T cells, producing INF-γ and TNF-α, were found after *M. hyopneumoniae* vaccination [12]. However, it is not completely clear how long *M. hyopneumoniae*-specific vaccine-induced immunity persists. In most *M. hyopneumoniae* vaccine efficacy studies, pigs are already challenged a few weeks after vaccination to investigate coughing, immune parameters, pathogen load, and lung lesions [13–16]. When the long-term efficacy was studied, mostly in field trials, often only production parameters and lung lesions at slaughter were examined and immune parameters were not or only poorly investigated [17–20]. To our knowledge, *M. hyopneumoniae*-specific vaccine-induced cell-mediated immunity from the moment of vaccination until slaughter under field conditions has not yet been investigated.

Besides piglet vaccination, *M. hyopneumoniae* vaccination of breeding gilts is a commonly used acclimation practice [21]. *Mycoplasma hyopneumoniae*-specific cytokine-producing CD4^+^CD8^+^ and polyfunctional CD4^−^CD8^−^ T cells are present after vaccination of breeding animals [22]. Piglets suckling colostrum from immunized sows have high levels of *M. hyopneumoniae*-specific maternally derived antibodies (MDA) and cell-mediated immunity in their blood which can persist till weaning or even longer [7, 22, 23]. When piglets are vaccinated against *M. hyopneumoniae* in the presence of (high levels of) MDA, seroconversion is often lacking [24–26]. However, the proliferation response of lymphocytes after in vitro stimulation was significantly higher in *M. hyopneumoniae* vaccinated piglets compared to non-vaccinated piglets, regardless of the levels of MDA [26]. Although these authors showed that the cell-mediated immunity was primed upon *M. hyopneumoniae* vaccination in the presence of high levels of MDA, further differentiation of the T cell subsets and their cytokine production is needed to gain better insights in these cell-mediated immune responses and eventually the influence of a natural infection on these responses.

The objective of the present study was to investigate the humoral and cell-mediated immune responses upon *M. hyopneumoniae* vaccination in pigs on two commercial farms. To this end, serum antibodies and proliferation of and cytokine production by different T cell subsets were monitored from the moment of vaccination at 16 days of age until slaughter age. The potential influence of *M. hyopneumoniae*-specific maternally derived immunity on humoral and cell-mediated vaccine responses in piglets was also investigated.

## Materials and methods

### Herd description and animal selection

Two commercial farrow-to-finish farms were included in the study based on the willingness of the farmer to participate. On both farms, circulation of *M. hyopneumoniae* was assumed based on the presence of the pathogen in tracheobronchial swabs (TBS) taken from fattening pigs in the past. Danbred breeding gilts were reared on farm A and purchased on farm B. On farm A, the breeding gilts were vaccinated once against *M. hyopneumoniae* four weeks prior to first insemination with Ingelvac MycoFLEX^®^ (Boehringer Ingelheim Vetmedica GmbH, Ingelheim am Rhein, Germany). On farm B, breeding gilts were vaccinated with Stellamune^®^ *Mycoplasma* (Elanco, Utrecht, The Netherlands) at six months of age upon arrival at the farm and a second time four weeks later. Furthermore, on farm B gilts were also booster vaccinated twice shortly before farrowing. On both farms, sows were not vaccinated against *M. hyopneumoniae*.

Farm A practiced a 5-week batch-farrowing-system and piglets were weaned and moved to the nursery unit at approximately 22 days of age. The piglets were vaccinated at 16 days of age against *M. hyopneumoniae* with an inactivated whole cell J strain-based bacterin (Ingelvac MycoFLEX^®^, Boehringer Ingelheim Vetmedica GmbH, Ingelheim am Rhein, Germany), porcine circovirus type 2 (PCV2) (Ingelvac CircoFLEX^®^, Boehringer Ingelheim Vetmedica GmbH) and porcine reproductive and respiratory syndrome virus (PRRSV) (UNISTRAIN^®^ PRRS, HIPRA, Amer, Spain). Pigs were moved to the fattening unit at 9 weeks of age.

Farm B worked in a 4-week batch-farrowing-system and piglets were weaned and moved to the nursery unit at approximately 22 days of age. They were vaccinated at 16 days of age against *M. hyopneumoniae* with the same vaccine as in farm A (Ingelvac MycoFLEX^®^, Boehringer Ingelheim Vetmedica GmbH) and PRRSV (UNISTRAIN^®^ PRRS, HIPRA). The piglets were moved to the fattening unit at 10 weeks of age. On both farms, five breeding animals (two gilts and three sows of mixed parity) were included in the study. The farrowing process was monitored by the main investigator and from each litter, five healthy piglets (birth weight >1 kg) were selected, ear notched and followed up monthly from birth till slaughter (n = 25 piglets / farm). Cross-fostering of the ear notched piglets was not allowed and pigs did not receive antibiotics active against *M. hyopneumoniae* on both farms during the entire trial.

### Study design and sampling

The study was approved by the Ethical Committee of the Faculty of Veterinary Medicine and the Faculty of Bioscience Engineering, Ghent University (approval number 2020 / 31). From the sows, colostrum was collected during the farrowing process. Within 6 h after farrowing, a TBS (60 cm sucking-catheter, Medinorm GmbH, Spiesen-Elversberg, Germany) and blood in a sterile serum tube (clotted blood) were collected from the sows. At two days of age, blood was taken from the piglets in serum tubes. From one to six months of age, blood in sterile serum and EDTA (non-clotted blood) tubes and a TBS were taken from the piglets on a monthly basis. On farm A, pigs were slaughtered at 177 days of age, and therefore, the last sample was taken at 5.5 months of age. On farm B, pigs were sent to the slaughterhouse at 191 days of age so the last sample was taken at 6 months of age. Colostrum and serum samples were stored at -20 °C and TBS samples at -80 °C until further analysis. Non-clotted blood was processed immediately for cell-mediated immunity analysis.

### Digital PCR for ***M. hyopneumoniae*** DNA detection

To test for the presence of *M. hyopneumoniae*, DNA was extracted from the TBS samples using a commercial kit (DNeasy^®^ Blood & Tissue kit, Qiagen, Venlo, The Netherlands) and a digital PCR (dPCR) protocol targeting the P102 gene was performed following a previously described protocol [27].

### ***Mycoplasma hyopneumoniae-*** specific antibodies

*Mycoplasma hyopneumoniae*-specific antibodies in serum and colostrum samples were analyzed using a commercial indirect ELISA (*M. hyo* Ab test, IDEXX Laboratories Inc., Westbrook, ME, USA) following the manufacturer’s instructions. Samples were considered positive if the sample to positive (S / P) ratio was higher than 0.40 and negative if the S / P ratio was equal to or lower than 0.40.

### T cell cytokine production

From the fresh, non-clotted blood, peripheral blood mononuclear cells (PBMCs) were isolated using a Lymphoprep™ density gradient (Stemcell technologies, Vancouver, Canada). Isolation, stimulation and staining of the PBMCs were performed as previously described with some minor modifications [22]. Cells were stimulated overnight (20 h) with in-house made *M. hyopneumoniae* J strain bacterin and for each animal a negative medium and positive Concanavalin A (ConA) control were included. The *M. hyopneumoniae* J strain bacterin was made based on the protocol for production of *M. hyopneumoniae* F7.2 C bacterin [16]. To investigate cytokine production, Brefeldin A (eBioscience™, San Diego, CA, USA) was added to each well for the last 4 h of stimulation to inhibit protein secretion. For extracellular staining, the PBMCs were incubated with anti-CD3 (clone PPT3), anti-CD4 (clone 74-12-4), and anti-CD8α (clone 11-295-33) monoclonal antibodies. Next, corresponding secondary antibodies anti-mouse IgG1 APC-Cy7 (Abcam, Cambridge, UK), anti-mouse IgG2b FITC (Biolegend, San Diego, CA, USA) and anti-mouse IgG2a PE-Cy7 (Abcam, Cambridge, UK) were added. Subsequently, a blocking step with mouse IgG1 (10 µg / mL) was performed. Following surface staining, cells were fixed, permeabilized and an intracellular staining for TNF-α, IFN-γ, and IL-17A was performed [22]. Data were acquired with a CytoFLEX flow cytometer (Beckman Coulter, Bea, CA, USA) and the results were further analyzed with CytExpert software (Beckman Coulter). The gating hierarchy is shown in Additional file 1.

### T cell proliferation assay

Isolated PBMCs were stimulated in vitro with in-house made *M. hyopneumoniae* J strain bacterin to assess proliferation of T cells. The protocol used was described in detail in a previous study [22]. For the surface staining, cells were first incubated with anti-CD3, anti-CD4, and anti-CD8α monoclonal antibodies and subsequently with the corresponding secondary antibodies anti-mouse IgG1 APC-Cy7 (Abcam, Cambridge, UK), anti-mouse IgG2b FITC (Biolegend, San Diego, CA, USA), and anti-mouse IgG2a AlexaFluor 647 (Biolegend, San Diego, CA, USA) together with propidium iodide. Data were acquired with a CytoFLEX flow cytometer and the results were further analyzed with CytExpert software. The gating hierarchy is shown in Additional file 2.

### Data analyses

Statistical analyses were performed using IBM SPSS^®^ Statistics Version 27 (IBM, Chicago, IL, USA) to compare results within farms. A descriptive analysis was performed for *M. hyopneumoniae*-specific antibody levels and the frequency of different T cell subsets. Kolmogorov-Smirnov and Shapiro-Wilk tests were used as tests for normality of the residuals. For normally distributed data, repeated measures analyses of variance were used and post-hoc pairwise comparisons were made with a Bonferroni correction to assess possible differences between successive sampling moments. For not normally distributed data, a non-parametric Friedman test was used together with a Wilcoxon signed rank test with Bonferroni correction to compare the different sampling moments.

Descriptive results were given to discuss whether the presence (S / P > 0.40) or absence (S / P ≤ 0.40) of MDA had an influence on the humoral and cell-mediated immunity after piglet vaccination.

## Results

On each farm, five pigs were excluded during the trial due to death or loss of ear tag. The data of these pigs were included in the analysis till the last correctly documented sampling moment.

### Presence of natural infection with ***M. hyopneumoniae*** on the farms

On farm A, all TBS samples (from sows, piglets and fattening pigs) tested negative for the presence of *M. hyopneumoniae* DNA. On farm B, all TBS samples from the sows, piglets and fattening pigs tested negative for the presence of *M. hyopneumoniae* DNA till five months of age. At six months of age, four fattening pigs tested positive for *M. hyopneumoniae* with 300, 7, 22 and 14 *M. hyopneumoniae* organisms/µL DNA.

### Persistence of ***M. hyopneumoniae***-specific antibodies in pigs

**Figure 1:**
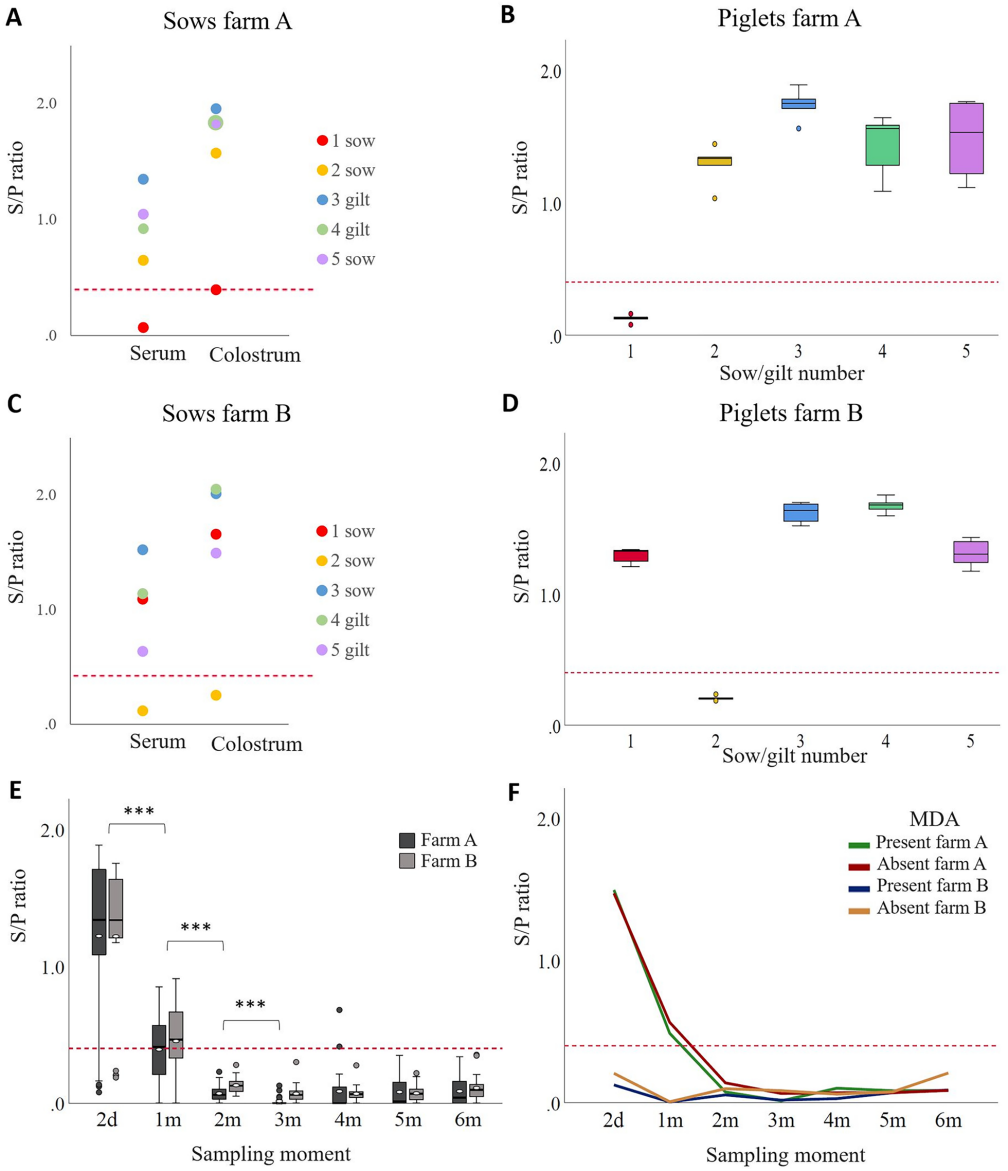
**Presence of** ***Mycoplasma hyopneumoniae***-**specific antibodies in sows and pigs**.** A, C** Presence of *M. hyopneumoniae*-specific antibodies in serum and colostrum of sows at farrowing; **B, D** Presence of *M. hyopneumoniae*-specific antibodies in serum of two-day-old piglets (5 piglets per sow). Color coding of the piglets corresponds to the color of their mother of which they ingested colostrum; **E** Persistence of *M. hyopneumoniae*-specific antibodies in serum of pigs at the different sampling moments on both farms; **F** Persistence of *M. hyopneumoniae*-specific antibodies in pigs with (S / P ratio > 0.40) and without (S / P ratio ≤ 0.40) *M. hyopneumoniae*-specific maternal antibodies at the different sampling moments on both farms. Pigs were vaccinated against *M. hyopneumoniae* at 16 days of age. ***, *P* ≤ 0.001 between the sampling moments. d: days, m: months, MDA: maternally derived antibodies; dashed red line = S / P ratio ≤ 0.40 are seronegative animals according to the manufacturer’s instructions of the commercial ELISA used.

On both farms, four out of five sows had *M. hyopneumoniae*-specific antibodies in serum and colostrum at farrowing (Figures 1A and C). Two-day-old piglets suckling colostrum from those sows had *M. hyopneumoniae*-specific maternal antibodies (MDA present), while piglets from seronegative sows were also seronegative (MDA absent) (Figures 1B and D). Despite vaccination at 16 days of age, on both farms, the level of *M. hyopneumoniae*-specific antibodies in serum of the pigs decreased significantly from two days of age to one month, from one month to two months, and from two months to three months of age (Figure 1E). No significant differences were seen between the later sampling moments. From two months of age onwards, all pigs, except two on farm A at four months of age, were *M. hyopneumoniae* seronegative. The *M. hyopneumoniae*-specific antibody levels from piglets with MDA decreased over time and at two months of age reached the same levels as those in piglets without MDA (Figure 1F).

#### Kinetics of T cell subset frequencies in pigs

**Figure 2:**
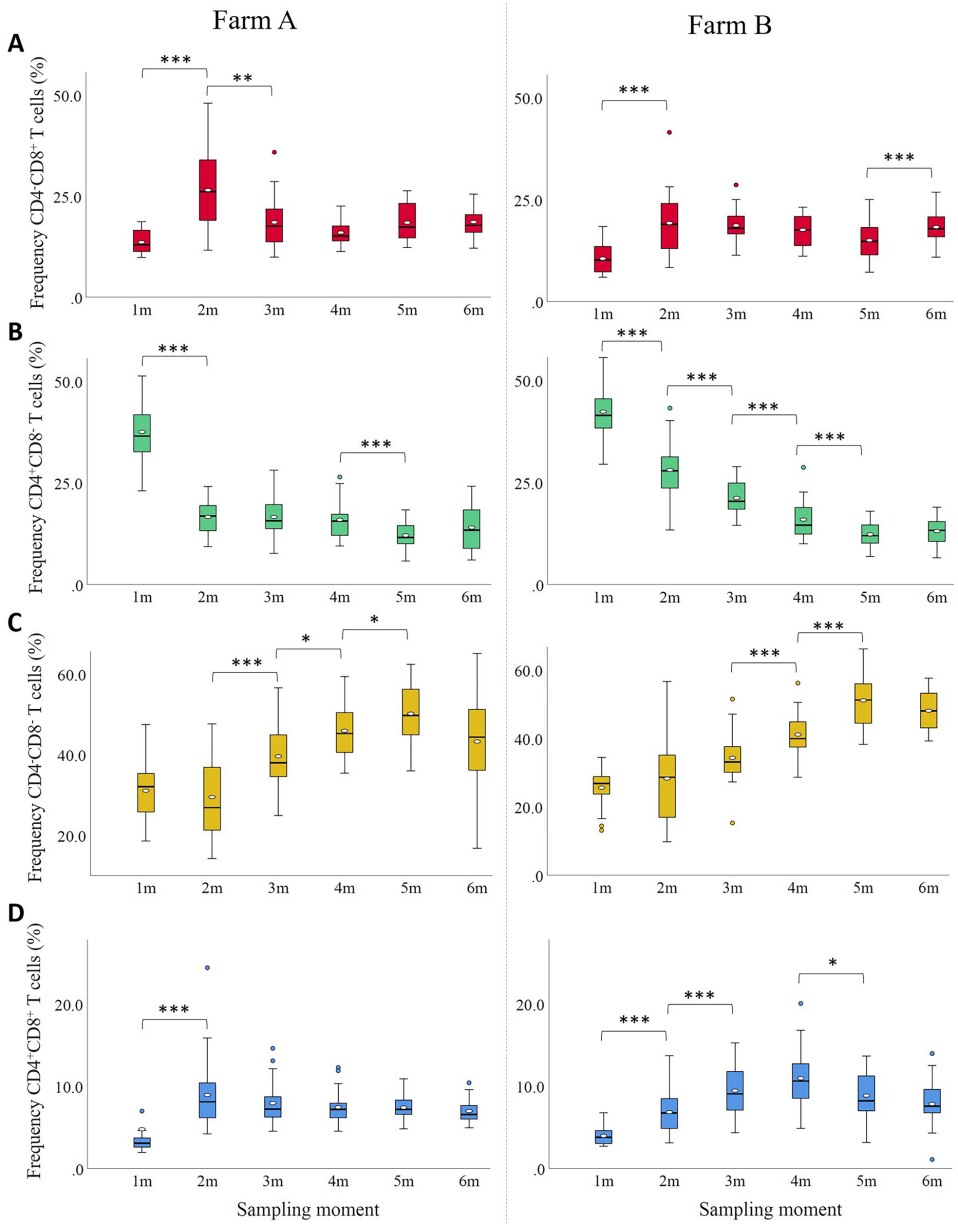
**Monthly frequency (%) of CD3**^**+**^** T cell subsets in blood of pigs**.** A** CD4^−^CD8^+^ T cells; **B** CD4^+^CD8^−^ T cells; **C** CD4^−^CD8^−^ T cells; **D** CD4^+^CD8^+^ T cells. Figures on the left: farm A, Figures on the right: farm B. On each farm, pigs were vaccinated against *M. hyopneumoniae* at 16 days of age. *, *P* ≤ 0.05; **, *P* ≤ 0.01; ***, *P* ≤ 0.001 between the sampling moments.

During the life of a pig, the frequency of the different T cell subsets in blood changes due to maturation of the immune system and contact with microorganisms [28]. To investigate the kinetics of the different T cell subsets, the blood PBMCs from the non-stimulated medium control were evaluated by flow cytometry. At one month of age, the frequency (average ± SD %) of CD4^−^ CD8^+^ T cells was 13.6 ± 2.8% and 10.5 ± 3.4% in pigs from farm A and B, respectively. This frequency significantly increased at two months of age on both farms, and declined again significantly at three months of age on farm A. From three months of age onwards it remained stable on farm A, while on farm B a significant increase was again observed at six months of age (Figure 2A).

On both farms, the frequency of CD4^+^CD8^−^ T cells was highest at one month of age with 37.5 ± 6.0% (farm A) and 42.3 ± 5.9% (farm B). This frequency significantly decreased at two months of age. On farm A, the frequency stabilized from two months onwards, while on farm B the decrease occurred more gradually (Figure 2B). At one month of age, the CD4/CD8 ratio was 2.8 (farm A) and 4.0 (farm B). The ratio dropped at two months of age and remained below 1.0 from three months of age onwards on both farms. The frequency of CD4^−^CD8^−^ T cells in one-month-old pigs was 31.1 ± 7.2% and 25.6 ± 5.4% on farm A and B, respectively. On both farms, the frequency remained similar at two months of age but significantly increased afterwards to reach the highest levels at five months of age (Figure 2C). The frequency of CD4^+^CD8^+^ T cells was lowest at one month of age with 3.3 ± 1.1% and 4.0 ± 1.0% on farm A and B, respectively. This frequency significantly increased at two months of age. On farm A, it remained stable afterwards, while on farm B it gradually increased further until four months of age to decrease again significantly at five months of age (Figure 2D).

#### Proliferation of T cells

**Fig. 3:**
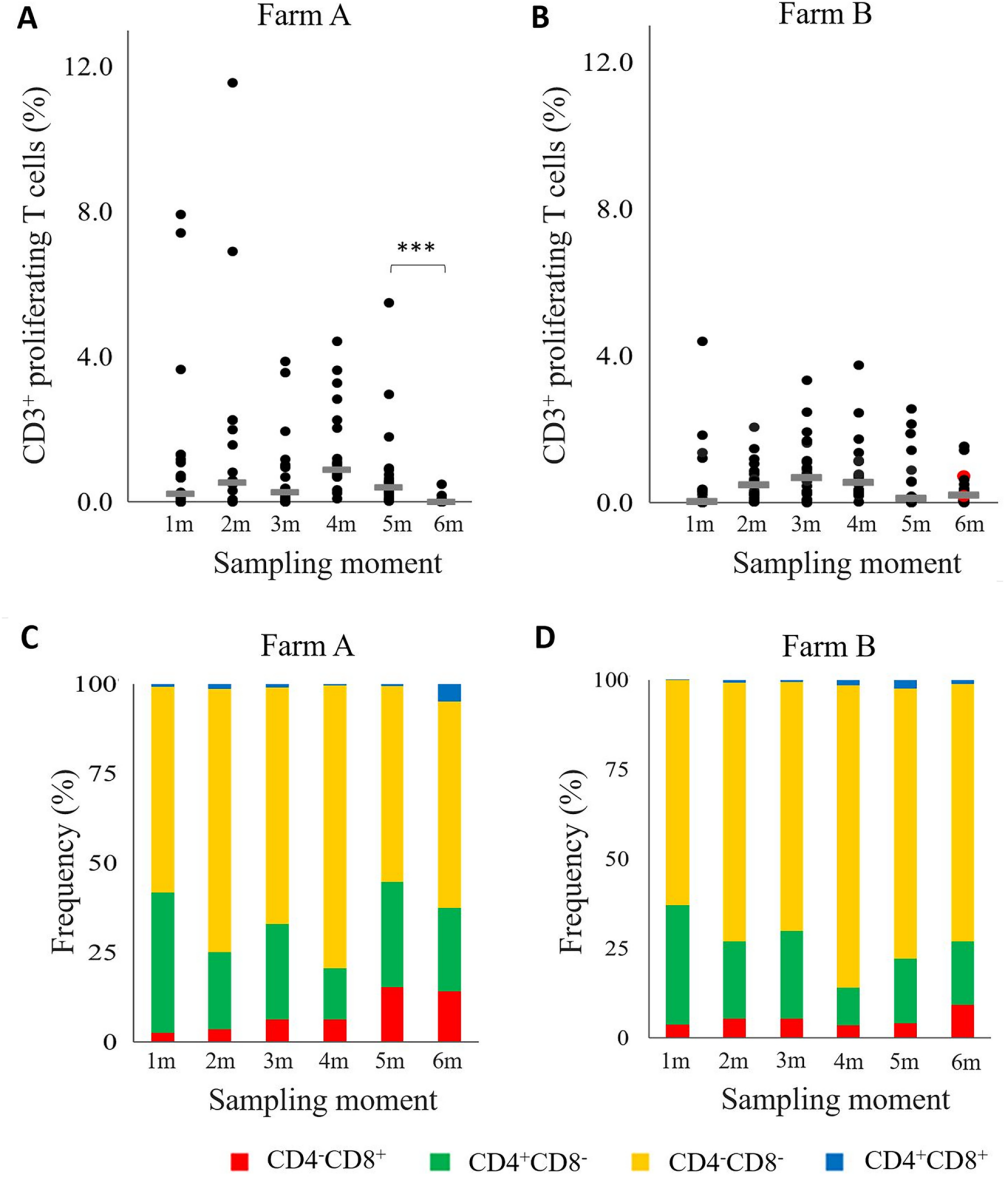
**Persistence of circulating proliferating CD3**^**+**^** T cells in pigs**.** A, B** Frequency (%) of proliferating CD3^+^ T cells on farm A and B, respectively. The horizontal grey line indicates the median **C, D** Frequency (%) of the proliferating T cell subsets in pigs at the different sampling moments. On each farm pigs were vaccinated against *M. hyopneumoniae* at 16 days of age. ***, *P* ≤ 0.001 between the sampling moments. Red dot: *M. hyopneumoniae* PCR positive animal.

The *M. hyopneumoniae*-stimulated PBMCs were analyzed to investigate the percentage of proliferating T cells. As shown in Figures 3A and B, proliferating CD3^+^ T cells were present in blood of pigs at each sampling moment on both farms after in vitro stimulation with *M. hyopneumoniae* bacterin, although the percentages of some animals were higher on farm A. On farm A, the percentage of proliferating T cells significantly decreased at six months of age as compared to the percentage at five months of age, while on farm B, no significant decrease or increase in the percentage of proliferating T cells could be observed. The CD4^−^CD8^−^ T cells were the dominant CD3^+^ T cell subset proliferating in response to *M. hyopneumoniae* stimulation on both farms, followed by the CD4^+^CD8^−^ T cell subset (Figures 3 C and D).

#### T cell cytokine production

**Figure 4:**
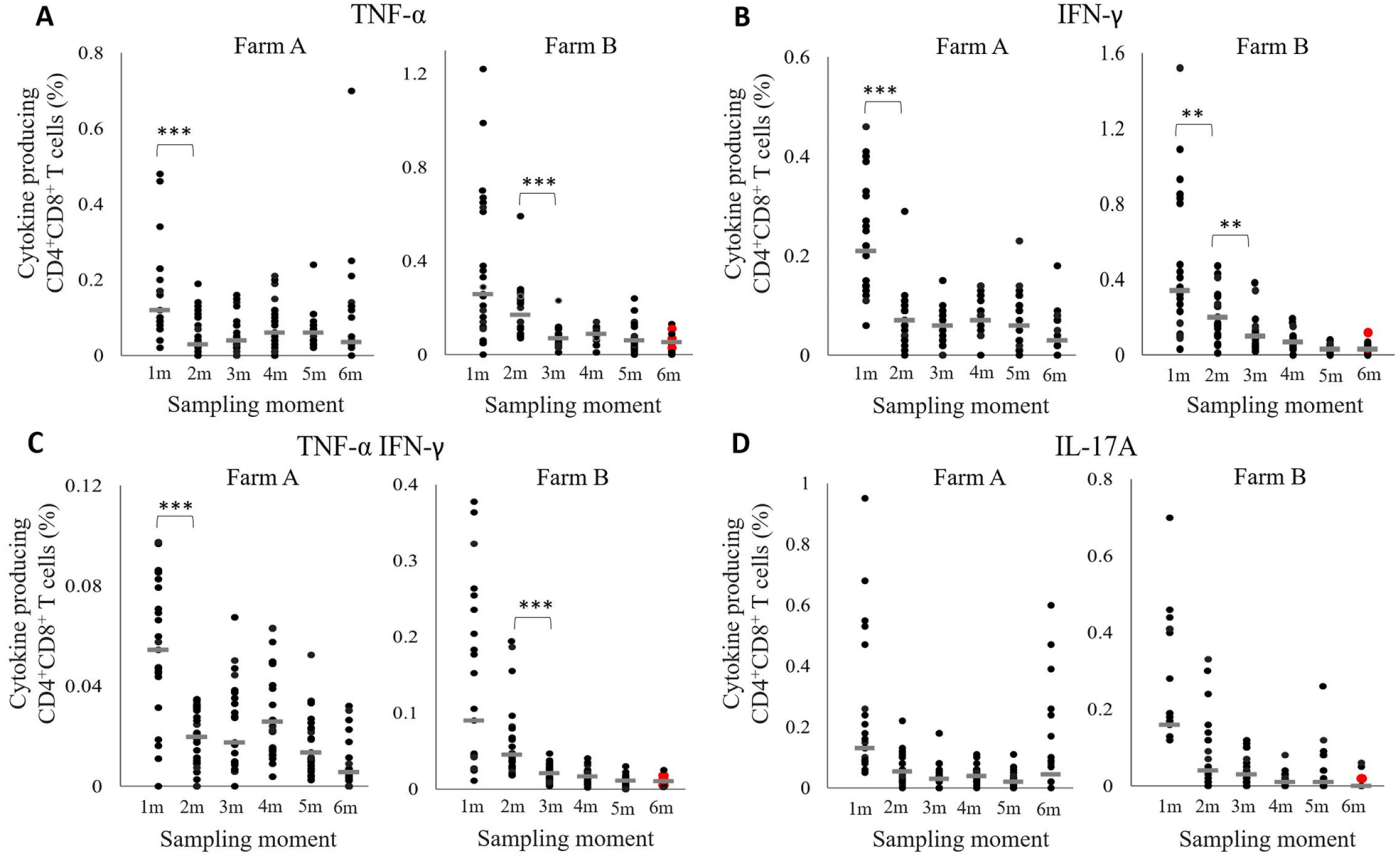
**Circulating** ***Mycoplasma hyopneumoniae***-**specific cytokine-producing CD4**^**+**^**CD8**^**+**^** T cells in blood of pigs on both farms. A** TNF-α; **B** IFN-γ, **C** TNF-α IFN-γ; **D** IL-17A. The horizontal grey line indicates the median. On both farms, pigs were vaccinated against *M. hyopneumoniae* at 16 days of age. PBMCs were stimulated with *M. hyopneumoniae* J strain bacterin and T cell phenotype and cytokine production were assessed by flow cytometry on a monthly basis. **, *P* ≤ 0.01; ***, *P* ≤ 0.001 between the sampling moments. Red dot: *M. hyopneumoniae* PCR positive animal.

**Figure 5:**
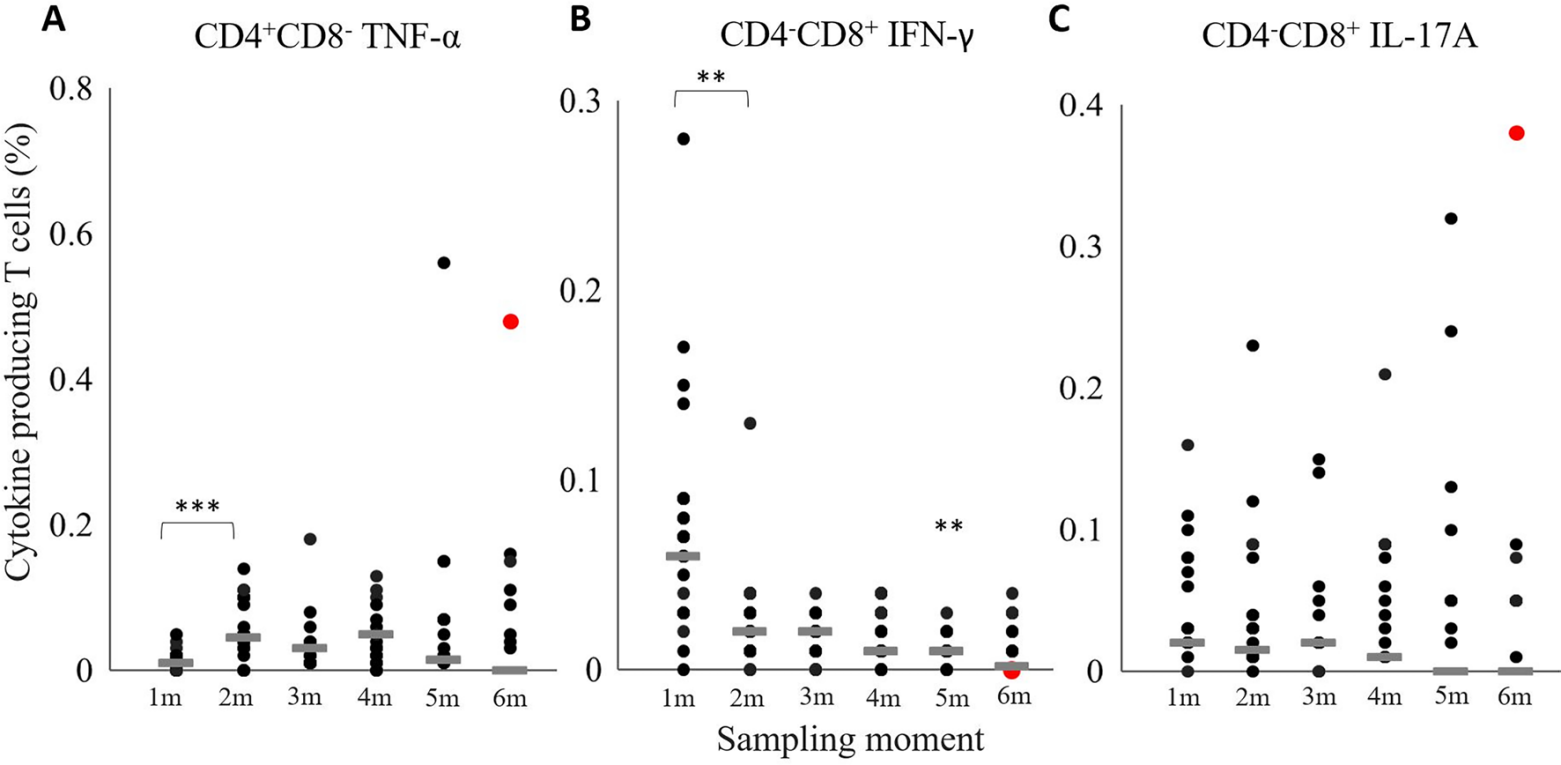
**Circulating** ***Mycoplasma hyopneumoniae***-**specific cytokine-producing T cell subsets in blood of pigs on farm B**.** A** CD4^+^CD8^−^ TNF-α; **B** CD4^−^CD8^+^ IFN-γ; **C** CD4^−^CD8^+^ IL-17A. The horizontal grey line indicates the median. On both farms pigs were vaccinated against *M. hyopneumoniae* at 16 days of age. PBMCs were stimulated with *M. hyopneumoniae* J strain bacterin and T cell phenotype and cytokine production were assessed by flow cytometry. **, *P* ≤ 0.01; ***, *P* ≤ 0.001 between the sampling moments. Red dot: *M. hyopneumoniae* PCR positive animal.

To gain a better insight in the presence and persistence of *M. hyopneumoniae*-specific T cells in pigs, TNF-α, IFN-γ and IL-17A production by blood PBMCs stimulated in vitro with *M. hyopneumoniae* bacterin were evaluated by flow cytometry. As shown in Figure 4, on both farms, stimulated CD4^+^CD8^+^ T cells produced cytokines, which was more pronounced in pigs of a younger age. The percentage of TNF-α^+^ CD4^+^CD8^+^ T cells decreased significantly in pigs on farm A from one to two months of age and from two to three months of age on farm B. A significant drop in IFN-γ^+^ CD4^+^CD8^+^ T cells was observed from one to two months of age in pigs on both farms, and from two to three months of age on farm B. The percentage of CD4^+^CD8^+^ T cells producing both TNF-α and IFN-γ decreased significantly in pigs from one to two months of age at farm A and from two to three months at farm B. At one month of age, the percentage of IL-17A^+^ CD4^+^CD8^+^ T cells was higher compared to the percentage at two months of age, but this difference was not statistically significant. The four animals on farm B that tested *M. hyopneumoniae* positive at six months of age had a similar cytokine production by CD4^+^CD8^+^ T cells as compared to the *M. hyopneumoniae* negative animals. In addition to the CD4^+^CD8^+^ T cell subset, we also observed differences in cytokine production by other T cell subsets on farm B. Figure 5 shows data of clear responses and significant differences in cytokine production by the other T cell subsets which only occurred in farm B. For the other parameters and on farm A, no differences were observed in the cytokine production of CD4^−^CD8^+^, CD4^+^CD8^−^ or CD4^−^CD8^−^ T cells. The percentage of TNF-α^+^ CD4^+^CD8^−^ T cells increased significantly, while the percentage of IFN-γ^+^ CD4^−^CD8^+^ T cells decreased significantly from one to two months of age. Of note, the pig that was *M. hyopneumoniae* positive at six months of age with 7 *M. hyopneumoniae* organisms/µL DNA had high percentages of TNF-α^+^ CD4^+^CD8^−^ and IL-17A^+^ CD4^−^CD8^+^ T cells in its blood. The other *M. hyopneumoniae* positive pigs at six months of age had similar percentages of cytokine-producing T cells than the *M. hyopneumoniae* negative pigs.

**Figure 6:**
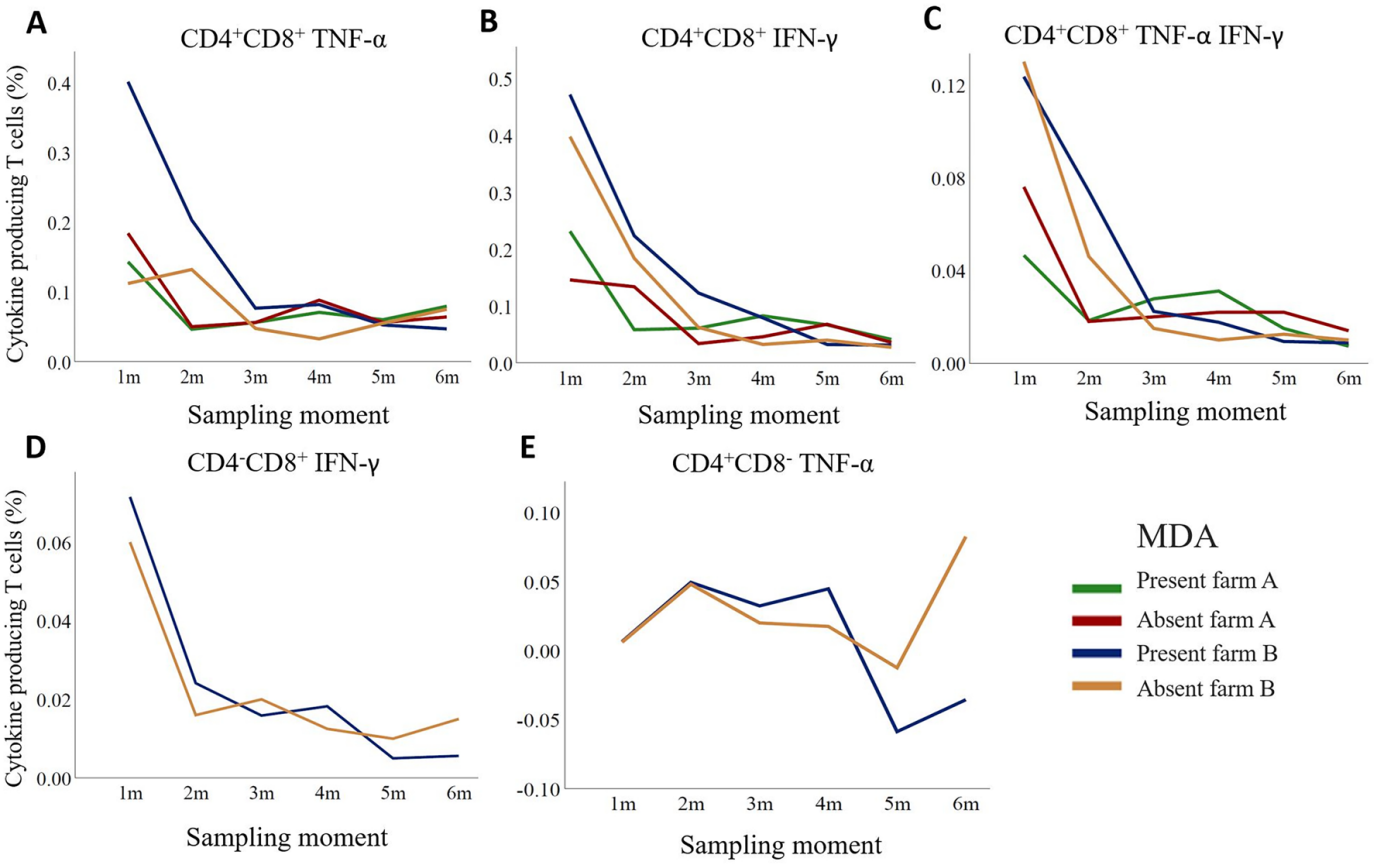
**Average percentage of** ***Mycoplasma hyopneumoniae***-**specific cytokine-producing T cell subsets by maternally derived antibody levels**.** A-C** Circulating *M. hyopneumoniae*-specific cytokine-producing CD4^+^CD8^+^ T cells in pigs on farm A and B; **D, E** cytokine-producing CD4^+^CD8^−^ and CD4^−^CD8^+^ T cells in blood of pigs on farm B (farm A is not shown, as there were no significant cytokine responses in CD4^+^CD8^−^ and CD4^−^CD8^+^ T cells seen in animals from farm A). Pigs were vaccinated against *M. hyopneumoniae* at 16 days of age on both farms. On each farm in 20 piglets *M. hyopneumoniae*-specific maternally derived antibodies (MDA) were present and in 5 piglets MDA were absent (present: S / P ratio > 0.40; absent: S / P ratio ≤ 0.40) at two-days of age.

On each farm, *M. hyopneumoniae*-specific MDA were present in serum of 20 two-day-old piglets and absent in serum of five piglets, which came from the sow without *M. hyopneumoniae*-specific serum antibodies. To investigate if MDA had an influence on the *M. hyopneumoniae*-specific vaccine-induced cell-mediated immunity, the distinction was made between the piglets with and without MDA, and differences between the two groups were investigated for the T cell subsets for which significant differences were observed in cytokine production (Figures 6A–E). On farm B, the percentage of TNF-α^+^ CD4^+^CD8^+^ T cells tended to be higher in one-month-old pigs with MDA, as compared to one-month old pigs without MDA. For the other cytokine-producing CD4^+^CD8^+^ T cells, the average percentages were similar between the two groups of pigs at one month of age, on each farm. Over time, there was a decreasing trend in average percentages of cytokine producing CD4^+^CD8^+^ T cells in blood of pigs with only small to no differences at six months of age between pigs with and without MDA (Figures 6A–C). The same was observed for IFN-γ-producing CD8^+^ T cells in blood of pigs on farm B (Figure 6D). At one and two months of age, the average percentage of TNF-α^+^ CD4^+^CD8^−^ T cells on farm B was similar for pigs with or without MDA, while at six months of age, a higher percentage of TNF-α-producing CD4^+^CD8^−^ T cells was observed in the blood of pigs without MDA (Figure 6E).

## Discussion

This is the first study providing insights in the long-term *M. hyopneumoniae*-specific vaccine-induced immune responses in pigs from birth till slaughter under field conditions. A humoral immune response in serum, assessed with the IDEXX ELISA, was absent after vaccination but cell-mediated immune responses were present. Proliferation of T cells after in vitro *M. hyopneumoniae* stimulation was observed till the end of the fattening period. Furthermore, at one month of age, after *M. hyopneumoniae* vaccination, *M. hyopneumoniae*-specific cytokine-producing CD4^+^CD8^+^ T cells, which include memory T cells, were present but decreased with increasing age. Maternal antibodies did not seem to affect the humoral and cell-mediated vaccine-induced immune responses during the life of the pig, except for the presence of TNF-α^+^ CD4^+^CD8^+^ T cells.

To evaluate the persistence and differences in immune response in *M. hyopneumoniae* vaccinated piglets at farm level, we included two commercial farms. Although circulation of *M. hyopneumoniae* was assumed on both farms, the studied pigs remained *M. hyopneumoniae* negative the entire trial, except four pigs on farm B at the end of the trial, which allowed us to investigate the persistence of the vaccine-induced immune responses without the influence of natural infection. The four *M. hyopneumoniae* PCR-positive pigs at six months of age on farm B did not have higher *M. hyopneumoniae*-specific antibody levels compared to the PCR-negative pigs. Except for one pig, they also did not have higher percentages of cytokine-producing T cells in their blood after in vitro *M. hyopneumoniae* stimulation. This could be explained by the fact that the *M. hyopneumoniae* infection likely occurred shortly before the moment of sampling, which did not allow the immune system sufficient time to respond to the infection [29–31]. In three out of the four *M. hyopneumoniae* positive pigs, the pathogen load in the TBS sample was also rather low.

Serum antibody levels in two-day-old pigs corresponded with the levels of *M. hyopneumoniae*-specific antibodies in serum and colostrum from sows at farrowing, confirming the transfer of *M. hyopneumoniae*-specific maternal immunity via colostrum [22, 23]. Seroconversion was not observed after *M. hyopneumoniae* vaccination at 16 days of age. On the contrary, levels of *M. hyopneumoniae*-specific antibodies decreased over time with all animals becoming seronegative at two months of age. The commercial *M. hyopneumoniae* vaccine used in this study is known to cause limited seroconversion after single administration, which was also observed in previous studies [14, 32, 33]. In an experimental study, vaccinating piglets around weaning, 40% of the animals had seroconverted 3 weeks post vaccination, while in a field trial only 3 out of 40 pigs seroconverted 7 weeks after vaccination [14, 32]. However, the presence (or absence) of *M. hyopneumoniae*-specific serum antibodies after vaccination does not correspond with protection (or a lack thereof) against infection [14, 25, 34].

To protect the host against facultative intracellular and extracellular bacterial pathogens like *M. hyopneumoniae*, CD4^+^CD8^−^ T helper cells, mainly Th1 and Th17 cells, play an important role. Th1 cells produce IFN-γ, which activates macrophages to kill the pathogen, while Th17 cells produce IL-17A to fortify the mucosal barrier [35–38]. Recently, it has been shown that a low percentage of *M. hyopneumoniae* cells can also be found intracellularly, and given the crucial role of CD4^−^CD8^+^ cytotoxic T cells in protecting the host against intracellular pathogens, these cytotoxic T cells might also be important to control *M. hyopneumoniae* infections [31, 35, 39]. To investigate the kinetics of the different T cell subset frequencies and the ability of these T cells to proliferate after in vitro *M. hyopneumoniae* stimulation, PBMCs were isolated from piglets on both farms. The pattern of T cell subset frequency was similar on these two farms. According to other studies, the percentage of CD4^+^CD8^−^ T cells is higher than the percentage of CD4^−^CD8^+^ T cells at a younger age. With increasing age, the percentage of CD4^+^CD8^−^ T cells decreases, while the percentage of CD4^−^CD8^+^ T cells increases resulting in a decreasing CD4/CD8 ratio over time [40–42]. Piglets are born with almost no CD4^+^CD8^+^ T cells. These cells increase with age when pigs have contact with microorganisms, as some of these cells have a pathogen-specific memory function [5, 22, 42–44]. However, on farm A, the frequency of CD4^+^CD8^+^ T cells remained at the same level from two months of age onwards. On farm B, there was a gradual increase till four months of age, like expected, although the percentage of CD4^+^CD8^+^ T cells decreased again afterwards. Comparing T cells subsets with other studies remains difficult as the PBMCs in this study were not measured directly after blood sampling, but first incubated for 20 h before measuring the percentage of T cell subpopulations. *Mycoplasma hyopneumoniae*-specific proliferation of CD3^+^ T cells persisted till the end of the fattening period, confirming the presence of *M. hyopneumoniae*-specific T cells in blood of pigs vaccinated at 16 days of age. The presence of *M. hyopneumoniae*-specific proliferating T cells might be correlated with protection against enzootic pneumonia because previous research demonstrated that the ability of PBMCs to proliferate, after in vitro stimulation with the mitogen ConA, is correlated with the resistance against diseases in pigs [45]. The CD4^−^CD8^−^ T cells were the main T cell subset proliferating on both farms followed by CD4^+^CD8^−^ T cells with the percentages fluctuating over time. In pigs, most CD4^−^CD8^−^ T cells bear invariant γδ T cell receptors in contrast to antigen-specific αβ T cells [31, 42]. Upon infection of pigs with other pathogens like PRRSV, γδ T cells were also the main proliferative responders post PRRSV viremia [46]. Those T cells also play a role in the production of neutralizing antibodies after vaccinating pigs against classical swine fever [47]. Furthermore, in bronchoalveolar lavage fluid from pigs infected with *Actinobacillus pleuropneumoniae*, an increase in CD8^−^ γδ T cells was observed [48]. Further research is necessary to investigate the role of γδ T cells in the control and protection against *M. hyopneumoniae* infections.

In recall assays, different T cell subsets were assessed for their production of TNF-α, IFN-γ and IL-17A. At two months of age, the percentage of TNF-α-, IFN-γ- and TNF-α/IFN-γ-producing CD4^+^CD8^+^ T cells were significantly decreased compared to the percentages at one month of age. Afterwards, the percentages were variable, they either decreased further or remained stable over time. The same pattern was seen for IL-17A-producing CD4^+^CD8^+^ T cells, although not statistically significant. In gilts vaccinated against PRRSV, IFN-γ production by PBMCs was also observed till 42 days post primo vaccination, but decreased afterwards till 147 days post-vaccination [49]. Increased numbers of IFN-γ secreting lymphocytes after *M. hyopneumoniae* vaccination has been demonstrated previously [9, 10].

Recently, evidence is accumulating that polyfunctional T cells, which produce more than one cytokine, have a pivotal role for protection and clearance of pathogens. For instance, in pigs infected with *Chlamydia* species, polyfunctional CD4^+^CD8^−^ T cells are correlated with protection [50]. Vaccination of piglets against *M. hyopneumoniae* also increased the percentages of polyfunctional CD4^+^CD8^−^ and CD4^−^CD8^+^ T cells [12]. In another study, vaccination of pigs against PCV2 resulted in higher levels of TNF-α^+^/IFN-γ^+^ CD4^+^CD8^+^ T cells at 24 days post-vaccination, decreasing again towards the end of the study at 56 days post-vaccination [51]. In our study, we also observed that the presence of cytokine-producing CD4^+^CD8^+^ T cells decreased over time. This study focused on circulating blood lymphocytes, but vaccination also induces local *M. hyopneumoniae*-specific cell-mediated immune responses in lungs and bronchial lymph nodes [8]. Therefore, future research on *M. hyopneumoniae*-specific vaccine-induced immunity should also focus on local cell-mediated immune responses. Furthermore, to immunize the piglets only one commercial *M. hyopneumoniae* vaccine was used in this study. The immune responses can differ between vaccines as they are influenced by the formulation of a vaccine (adjuvant and antigen) and the administration route [16, 52, 53]. For example, carbopol, the adjuvant in the commercial *M. hyopneumoniae* vaccine used, is known to direct the immune response towards a Th1 response [54].

On each farm, five piglets were *M. hyopneumoniae* seronegative and in serum of 20 piglets *M. hyopneumoniae*-specific MDA were present. Regardless of their MDA status, all pigs were *M. hyopneumoniae* seronegative at two months of age and seroconversion was not observed at later time points. Our findings correspond with a previous study in which seven-day-old piglets with and without MDA were vaccinated against *M. hyopneumoniae* [25]. In both groups, seroconversion after vaccination was not observed and all pigs were seronegative at 42 days post vaccination. On the other hand, others found that *M. hyopneumoniae* vaccination in the presence of MDA resulted in higher antibody levels compared to non-vaccinated piglets, but the higher the titer of MDA, the lower the response [24, 26]. Although an antibody response is not always observed after *M. hyopneumoniae* vaccination, *M. hyopneumoniae*-specific cell-mediated immune responses are induced [26]. No difference was observed in proliferation of PBMCs isolated from *M. hyopneumoniae* vaccinated pigs regardless of the levels of MDA [26]. In our study, within each farm, no differences were observed in cytokine-producing T cells between *M. hyopneumoniae* vaccinated pigs with and without MDA at one month of age. Only on farm B, piglets with MDA tended to have higher percentages of TNF-α^+^ CD4^+^CD8^+^ T cells at one month of age compared to piglets without MDA. The relevance of these findings should be further investigated in a study including a larger and equal amount of piglets with and without *M. hyopneumoniae*-specific MDA. Then also statistical analysis should be performed as this was not done in this study due to the low and unequal number of animals in the group without and with MDA.

In conclusion, the present study showed that, although seroconversion after *M. hyopneumoniae* vaccination of piglets was lacking, *M. hyopneumoniae*-specific cell-mediated immune responses were detected. Polyfunctional cytokine-producing CD4^+^CD8^+^ T cells were present till three months of age and proliferating T cells were observed till the end of the fattening period indicating the presence of a pathogen-specific cell-mediated immunity in *M. hyopneumoniae* vaccinated pigs. The results form the basis for further research assessing the influence of a natural *M. hyopneumoniae* infection on the vaccine-induced immunity under field conditions.

## Supplementary Information

**Additional file 1.** Gating strategy to assess cytokine production by T cells with CytExpert software.

**Additional file 2.** Gating strategy applied on the T cell proliferation assay with CytExpert software.

## Abbreviations

αβT cells: alpha beta T cells
ConA: Concanavalin A
γδT cells: gamma delta T cells
IFN-γ: interferon gamma
Ig: immunoglobulin
IL-17A: interleukin 17 A
MDA: maternally derived antibodies
*M. hyopneumoniae*: *Mycoplasma hyopneumoniae*
dPCR: digital polymerase chain reaction
PBMCs: peripheral blood mononuclear cells
PCV2: porcine circovirus type 2
PRRSV: porcine reproductive and respiratory syndrome virus
SD: standard deviation
S / P ratio: sample to positive ratio
TBS: tracheobronchial swab
TNF-α: tumor necrosis factor alpha
