# Supplementary figures and images for: Long-term follow-up of Mycoplasma hyopneumoniae-specific immunity in vaccinated pigs
Source: Vet Res. 2023 Mar 1;54:16. doi: 10.1186/s13567-023-01145-1 (PMC9979462; doi:10.1186/s13567-023-01145-1)

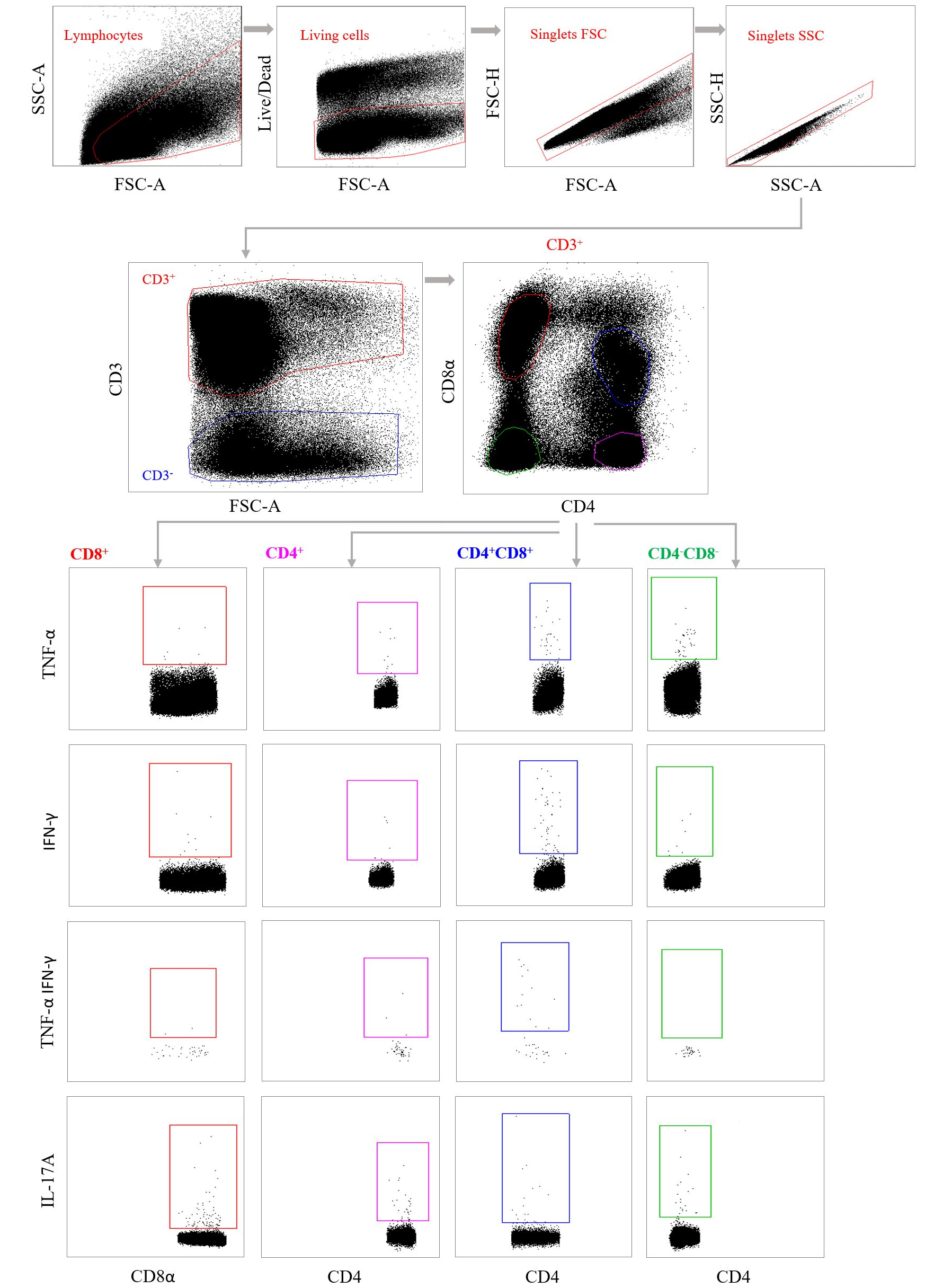

Supplement: Supplementary file 1 — Additional file 1. Gating strategy to assess cytokine production by T cells with CytExpert software. [file 13567_2023_1145_MOESM1_ESM.jpg]

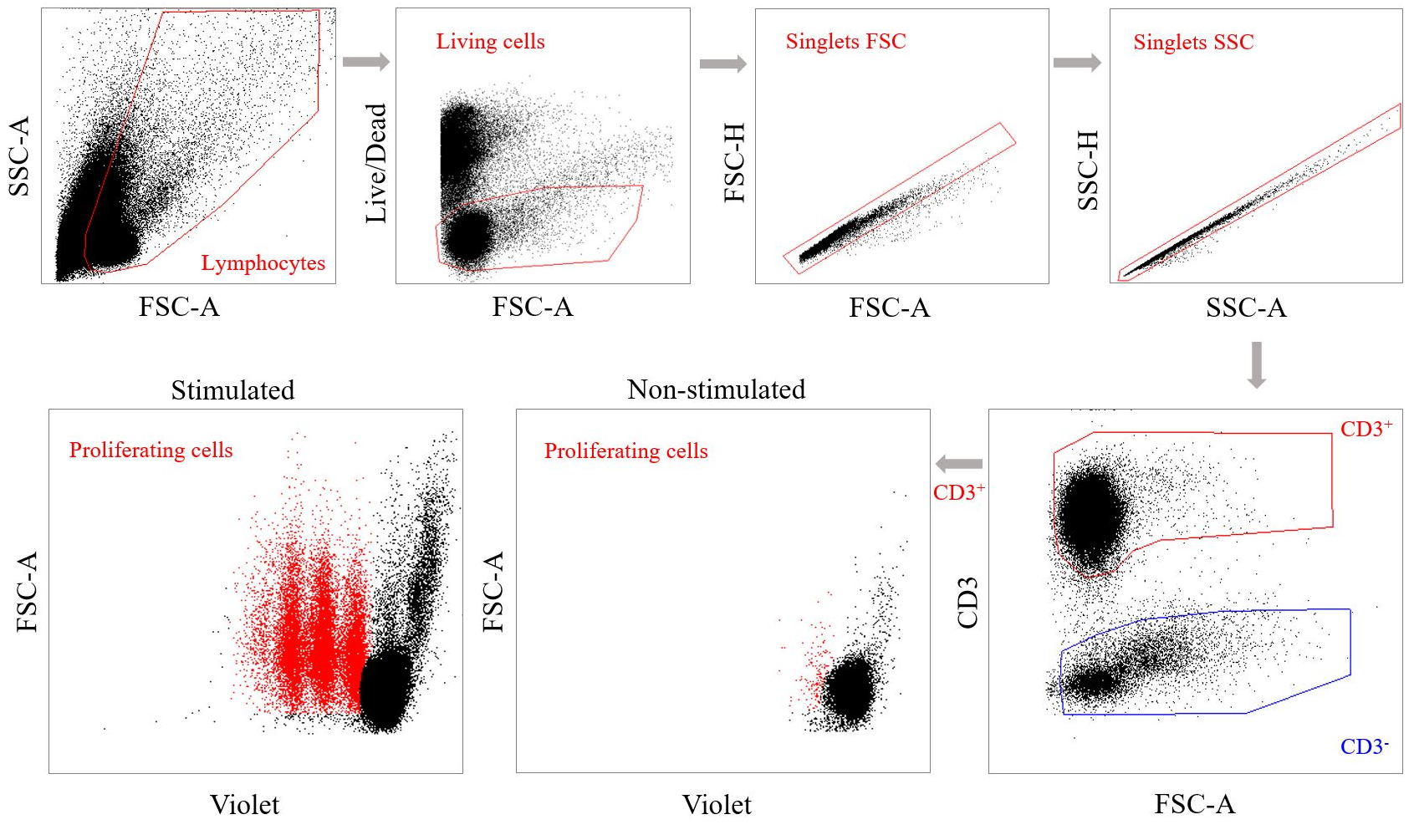

Supplement: Supplementary file 2 — Additional file 2. Gating strategy applied on the T cell proliferation assay with CytExpert software. [file 13567_2023_1145_MOESM2_ESM.jpg]
